# Educational-Care Needs for Hospital–Home Transition of Children With Hematological Cancer

**DOI:** 10.1155/nrp/4551422

**Published:** 2025-09-02

**Authors:** Thais Maia Teixeira Vieira, Liliane Faria da Silva, Fernanda Garcia Bezerra Góes, Tania Vignuda de Souza, Michelle Darezzo Rodrigues Nunes, Juliana Rezende Montenegro Medeiros de Moraes

**Affiliations:** ^1^Anna Nery School of Nursing (EEAN), Universidade Federal do Rio de Janeiro (UFRJ), Rua Afonso Cavalcanti, 275, Cidade Nova, Rio de Janeiro, Brazil; ^2^Aurora de Afonso Costa School of Nursing (EEAAC), Universidade Federal Fluminense (UFF), Rua Dr. Celestino, 74-Centro, Niterói, Rio de Janeiro, Brazil; ^3^Universidade Federal Fluminense (UFF), Rua Recife, Quadra 7, Lotes de 1 a 7, Jardim Bela Vista, Rio das Ostras, Rio de Janeiro, Brazil; ^4^Department of Maternal and Child Nursing, Faculty of Nursing, Universidade do Estado do Rio de Janeiro (UERJ), Boulevard 28 de Setembro, 157-Vila Isabel, Rio de Janeiro, Brazil

**Keywords:** child, health education, oncology, transition from hospital to home

## Abstract

**Background:** The transition from hospital to home is a critical period for families of children with cancer, requiring adaptation and new knowledge acquisition to manage treatment at home. Lack of adequate preparation can generate insecurity and negatively impact the quality of home care.

**Objective:** To describe the educational and care demands of family members of children with cancer during the hospital–home transition. By addressing these needs, this study seeks to contribute to health education practices which facilitate a safer and less stressful transition for families.

**Methods:** This is a qualitative and descriptive study developed through semistructured interviews from July to October 2023, with 20 family members of children with cancer undergoing chemotherapy treatment in a public pediatric hospital located in Brazil. The data were processed in Iramuteq software, organized by descending hierarchical classification for lexical analysis and interpreted in light of Afaf Meleis' transitions theory.

**Findings:** Two thematic blocks were structured related to the guidance required by family members and strategies for home care, as well as guidance shared by nurses for the hospital-to-home transition. The educational-care demands included the need for guidance.

**Conclusion:** The study demonstrated the need for nursing actions related to guidance for families on the care to be developed after discharge, with the aim of helping them deal with stressful situations, challenges, and difficulties associated with the hospital-to-home transition process. Nurses should be attentive to the educational-care demands of families, recognizing their particular difficulties and promoting collaborative, welcoming, and healthy learning environments.

## 1. Background

Developing countries have seen significant changes in their epidemiological profile in recent decades, characterized by a decrease in infectious and parasitic diseases and an increase in chronic noncommunicable diseases. Neoplasms stand out among these conditions as one of the main causes of mortality, reflecting a transition in the public health pattern and generating new challenges for health systems [1, 2].

Approximately 400,000 children and adolescents between the ages of 0 and 19 are diagnosed with cancer each year worldwide. It is estimated that at least 29,000 children and adolescents in the Americas and the Caribbean will be affected by the disease annually, resulting in almost 10,000 deaths. Childhood cancer cannot be prevented, but more than 80% of children diagnosed with cancer in high-income countries are cured, while the cure rate in many low- and middle-income countries is approximately 20% [3].

In this epidemiological context, childhood cancer has emerged as an important cause of death among children. However, technological advances aimed at early diagnosis and treatment have provided cure rates that are approaching 80% [4, 5].

Childhood cancer treatment is long and involves painful procedures which impact the lives of the child and their family. It is mostly performed in a hospital setting through hospitalization in specialized centers and also on an outpatient basis, which enables the transition from hospital to home during treatment. Treatment involves situations of separation from friends, family, and school, as well as changes in the child's social routine and daily life. Therefore, it is necessary to implement measures which ensure care continuity in all treatment phases, at which time family members assume a strategic role [6, 7].

The transition process from hospital to home care in children with cancer must be planned and systematic from the moment of diagnosis and commencement of treatment, so that nurses can effectively guide family members. Children and family members remain in a continuous flow of comings and goings to the hospital during cancer treatment. This experience is generally permeated by uncertainty and feelings of anxiety, stress, insecurity, and expectations of a cure, because everything is new and the treatment is challenging [8, 9]. Returning home should also involve the school and the community, mitigating difficulties in promoting and maintaining care and possible clinical complications [10, 11].

From this perspective, the transition theory developed by Afaf Meleis is suitable as a theoretical framework, since it describes typologies of transitions in terms of health–illness and situational. The health–illness transition is characterized by the change from a state of well-being to one of illness. On the other hand, the situational transition refers to specific events that impact changes in roles and contexts, directly influencing family and social dynamics [12].

Studies indicate that transition concepts in the nursing area have been underutilized, being more frequently applied in the occupational therapy and social work areas. However, care transitions have a significant impact on the health outcomes of children and families, in addition to directly influencing the care experience [13]. The hospital–home transition is marked by health–illness and situational transitions, and is particularly relevant and necessary for family members of children with cancer, especially to identify their educational demands in the care process. In this context, the transitions theory can be a valuable tool for nurse educators by enabling the development of appropriate interventions that support families in home care [14, 15].

The problems faced by caregivers of children with cancer in the hospital–home transition include a lack of clear information about treatment management, difficulties in adapting to the new demands of home care, and emotional and physical overload. The absence of systematic and personalized educational interventions increases the risk of clinical complications and hospital readmissions [13, 15]. Thus, this study is justified by an identification of gaps in support practices offered to family members of children with hematologic cancer during the hospital–home transition, seeking to produce evidence for developing effective, individualized, and targeted interventions in order to achieve better clinical results, well-being, and quality of life for all involved [12–16].

In view of the above, the objective of this study was to describe the educational and care demands of family members of children undergoing cancer treatment in the hospital–home transition.

## 2. Methods

### 2.1. Study Design and Setting

This is a descriptive study with a qualitative approach developed through semistructured interviews. The setting was a public pediatric hospital belonging to the Brazilian Unified Health System (*Sistema Único de Saúde*—*SUS*), a reference for treating children with special health needs, including hematologic cancer, located in the city of Rio de Janeiro (Brazil). Children access the hospital for childhood cancer treatment through the emergency department, the institution's own outpatient clinic, or by referral from basic health units through a computerized system for regulating vacancies of the Brazilian *SUS*. This health institution serves children from 1 month to 13 years of age.

### 2.2. Participant Eligibility Criteria

The inclusion criteria for participants were: family members over 18 years of age; for children, aged zero to 13 years in the induction, consolidation, or maintenance phase of treatment for hematologic cancer, who had already returned home after the initial diagnosis. The exclusion criteria were: family members who did not participate in the home care of the child, those with difficulties in verbal communication and who did not speak Portuguese, and family members of children with relapse of the disease and/or in palliative care. “Family” is understood in this study to be the mother, father, siblings, grandparents, neighbors, or friends (among others), since their meanings are defined by bonds of affection, intimacy, and love, and not only by blood kinship and the legal systems that govern family relationships [17].

After reading the child's medical records and completing the participant selection instrument, 23 potential participants were approached and invited personally and privately; however, three refused to participate, totaling 20 study participants.

### 2.3. Data Collection

The interview was conducted from July to October 2023, in person by the main researcher, in a private room at the hospital itself. The interviews lasted an average of 20 min and were developed using a script consisting of 10 questions regarding characterization of the family member and three more about childcare, totaling 13 questions. The interview script included questions about sociodemographic data, such as degree of relationship with the child, age, sex, race/color, religion, family income, marital status, receipt of social benefits, and availability of help for home care of the child. In addition, open-ended questions were asked about the child's hospital–home transition. All interviews were digitally recorded in audio and later transcribed by the main researcher to ensure the greatest possible fidelity to the manifest content. All participants who agreed to participate in the study signed an informed consent form (ICF), guaranteeing their anonymity and other ethical provisions contained in the resolutions of the National Health Council of the Ministry of Health (Brazil) [18]. The study was approved by the Research Ethics Committees of the São Francisco de Assis Teaching Hospital and the Anna Nery School of Nursing (Opinion No. 6,073,868), as well as the Research Ethics Committee of the Martagão Gesteira Institute of Childcare and Pediatrics (Opinion No. 6,175,320). The transcribed content was returned for reading by the participants, who did not feel the need to include or exclude any other data.

Data collection ended when the sample number of participants who met the eligibility criteria was saturated; theoretical saturation was defined based on information repetition (manifest content) about the research objective, and an 87.5% usage rate in the text processing software. Repetition of the response pattern was identified from the 17th interview onwards, and then three more participants were interviewed to in fact attest to the sampling and theoretical saturation [19].

### 2.4. Data Processing and Analysis

After obtaining the textual corpus from the interviews, the data were processed using Iramuteq software (*Interface de R pour les Analyses Multidimensionnelles de Textes et de Questionnaires*) in version 0.7 alpha 2, available on the website responsible for promoting the software (https://www.iramuteq.org/).

Iramuteq contemplates a set of distinct possibilities for processing qualitative data based on lexicometry. Thus, the descending hierarchical classification (DHC) was chosen for this study, which allows structuring text segments (TS), grouping the corpus vocabularies that present similarities between them, which then enables presenting the data in classes and the relationships between them.

It is noted that the development of this study met the recommendations of the Consolidated Criteria for Reporting Qualitative Research (COREQ).

## 3. Results

### 3.1. Demographics

Of the 20 family members, 15 (75%) were mothers and 5 (25%) were fathers, aged between 18 and 56 years. Regarding formal education, 4 (20%) had completed elementary school, 12 (60%) completed high school, and 4 (20%) had higher education.

The treatment phases in which the children were at the time of the interview were as follows: five (25%) were in the induction phase, eight (40%) in the consolidation phase, and seven (35%) in the maintenance phase of treatment. The children's ages ranged from 1 to 12 years, with an average of 7.5 years. Their diagnoses were acute lymphocytic leukemia, representing 15 cases (75%), followed by acute myeloid leukemia with three cases (15%) and lymphomas with two cases (10%).

### 3.2. Emergent Themes

**Figure 1:**
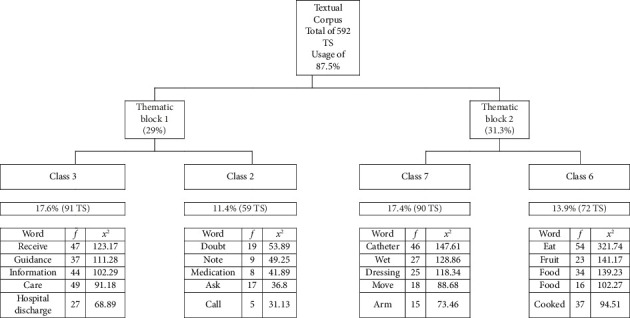
Structured dendrogram based on the textual corpus of interviews with family members. Source: adapted from the dendrogram generated by Iramuteq software (Version 0.7 alpha 2).

Processing the textual corpus by the Iramuteq software presented the following results: 20 texts, 7 classes, 592 TS, 2182 forms, 20,608 occurrences, 1119 active forms, 184 supplementary forms, and 518 TS classified from 592 TS (with a utilization of 87.5%). Thus, Classes 3, 2, 7, and 6 were considered for analysis because they contain content closer to the objective outlined in this study (see Figure 1).

#### 3.2.1. Thematic Block 1: Guidelines Required by Family Members and Strategies for Home Care

This block consisted of Classes 3 and 2. In Class 3, family members highlighted the need to receive guidance and information to care for their child at home. However, due to the large amount of information, they needed time to understand the care related to feeding, treatment phases, activities allowed during treatment, management of the long-term catheter at home, issues related to their children's school, the purpose of the exams, and information about the disease.

In this sense, they would like to receive this guidance in writing as a source of information for consultation at home and to share with the child's other caregivers in order to assist in the care provided to their children.

There's a lot to learn at first. It takes a while for you to understand, even after several explanations. That's why I think that if these instructions on how I'm going to take care of my son at home, with exams, what they're for, what to do at each stage of treatment, feeding, how to take care of the catheter, what can and can't be done, were in writing, it would help a lot. (Fam_19).

The information I would have liked to have received was in relation to feeding, as I wasn't given any guidance on or don't remember being given any. I did what I thought was right. (Fam_11)

[…] He [the child's father] doesn't ask. He's embarrassed. He gets home and doesn't know what to do. […] If there was something written down explaining everything I heard about the catheter, food, school, the disease, these basic guidelines for home care, it would be great, because he [the father] would read it and would be one more person to help me. (Fam_08)

[…] Taking care of my child after hospital discharge was challenging at first. […], at the same time, there were several guidelines and care instructions that we learned in a short time and needed to put into practice, and there was nothing written down. (Fam_20)

In Class 2, family members reported difficulties in assimilating the large amount of information received. Upon returning home, they used some strategies to clarify their concerns, including questions about the child's response to medications, possible reactions to chemotherapy, the next treatment stages, permitted and prohibited medications, blood collection, dressings, and care for the long-term catheter.

The strategies adopted by families included writing down questions that arose at home so that they could be clarified during future appointments and carefully observing the care provided at the hospital in order to reproduce them at home.

It was hard for me to absorb this large amount of information. It's impossible to assimilate everything. So, as I went back for appointments, blood collections and dressings, I kept asking questions. I wrote everything down at home and brought my questions back here to ask. (Fam_06)

[I had questions] about the disease, fear of relapse, whether he was responding well to the medications, the reactions he might have with the chemotherapy he takes every week, what the next stages of treatment are […]. (Fam_17)

When she was hospitalized, I observed how the care was given so I could do it at home. So, I had questions about the catheter, chemotherapy, medications, what I could give, what I couldn't… There was a lot of medication at the beginning. I asked the nurse, the doctors, and they explained it to me. That helped me a lot in the beginning. (Fam_05).

#### 3.2.2. Thematic Block 2: Guidelines Provided by Nurses for Home Care of Children Undergoing Hematological Oncology Treatment

This second thematic block was composed of Classes 7 and 6. In Class 7, family members highlighted the guidance received from nurses on care for the long-term catheter at home with the aim of avoiding infections and catheter loss.

The guidance included regular scheduling for dressing the long-term catheter, avoiding scratching, pulling, straining, or forcing the arm where the catheter is inserted, as well as restrictions on physical activities such as cycling, jumping, running, sunbathing, swimming in a pool, and getting the catheter wet. It was observed that the specific care required for the long-term catheter at home restricts the child's social activities and significantly influences their way of life.

The nurse said that he couldn't scratch, he couldn't make any effort, he couldn't strain his arm. He couldn't ride a bike, run, jump, sunbathe, or swim in the pool, because he could get an infection and lose the long-term catheter. (Fam_09).

The nurse gave me instructions and explained how to take care of my child when they showered, not to get it wet, not to pull it, not to move the catheter […] (Fam_05).

The day before the long-term catheter was inserted, the nurse went to the ward and gave us instructions on how to take care of the catheter when he went home. They scheduled his return appointment to do the dressing. (Fam_08).

In Class 6, family members pointed out the guidelines they received regarding the diet after the diagnosis and start of chemotherapy treatment, such as eating only cooked or baked foods, avoiding fried foods, not eating out, prioritizing eating at home and if going out, bringing what you want to eat, not eating foods you do not know where they come from, not eating any raw foods, and only eating fruits with thick skin and preferably cooked. In this sense, the change in the child's eating habits also led to the need to readjust the diet of the entire family.

[…] She only eats cooked foods now. She had to readjust her diet. We didn't eat that all the time, only sometimes. But now, not even that. (Fam_02)

[…] At home, everything used to be fried. Now, everything is baked. We only cook what she can eat. So, the family's diet has changed too. (Fam_12)

[…] He was told not to eat out, to prioritize eating at home, because we don't know where it comes from. Take whatever he's going to eat with him when he goes out. Don't eat anything raw, only things that can be cooked. Fruit, only with thick skin, preferably cooked. (Fam_13)

[…] Try to give him healthy foods […]. His diet was what worried me the most, because I wanted him to eat healthier. But he doesn't accept it well, he complains. (Fam_19)

## 4. Discussion

It is clear in the statements of family members of children undergoing cancer treatment that the educational and care demands in the hospital–home transition are partially met by nurses and other professionals from the multidisciplinary team, because even if family members receive information and guidance for home care, these take time to be internalized and family members still have uncertainty. Furthermore, information during the hospital–home transition is mostly transmitted orally, without the support of visual, educational, or explanatory resources, which makes it difficult for family members to assimilate the transmitted information [11, 12].

The interviewed families requested further information about administering medications, reactions to chemotherapy, caring for long-term catheters, hygiene procedures and adequate nutrition during treatment, performing exams and dressings, as well as the need for support to deal with constant uncertainty about the disease, managing symptoms and adverse reactions to chemotherapy treatment, as well as treatment phases and care when returning to school activities.

The literature corroborates the participants' statements, since family members often take responsibility for providing care in order to ensure care continuity in the home environment. Therefore, it is essential that the information shared by health professionals occurs systematically and in a time frame that is appropriate for effective understanding. In turn, planning and preparing for hospital discharge emerge as essential aspects of nursing care, aiming at a safe and effective transition to home [20, 21]. Furthermore, active interaction between family members and health professionals plays a relevant role in this process. This interaction not only facilitates access to clear and objective information but also enables clarifying questions and strengthens the self-confidence necessary for safe and effective management of childcare in the home environment [12].

Inhibiting conditions related to the care transition were identified based on Afaf Meleis' transition theory and the participants' statements, which can be classified as personal, community, and social [12]. In the context of this study, the personal inhibiting conditions included embarrassment on the part of some family members in clarifying concerns and questions, difficulty in assimilating a large amount of information transmitted, and lack of emotional preparation to deal with the situation. Among the community inhibiting conditions, the absence of written educational materials for home consultation, transmission of guidelines in a limited way or to only one family member, and lack of practical support in the home context were observed. In addition, the social inhibiting conditions included changes in the family's eating habits that impact the social routine, impact on the child's social activities, and lack of an external support network for care. Research highlights the importance of the multidisciplinary team being involved in providing discharge guidance, recognizing the fundamental role of the nurse as a link and educator with families, and facilitating identification of possible difficulties faced by them in health–disease transition situations, as was pointed out in the statements of participants in this study [21–23].

Based on the interpretation of the participants' statements, we start from the premise that nurses are configured as important agents in training actions for the hospital–home transition by promoting learning and experiencing new experiences. This can positively influence the adaptation, health promotion, and quality of life of children and their families during the transition process [12].

However, nurses may encounter difficulties in implementing comprehensive educational and care actions due to the multiple responsibilities they assume in care practice. This can negatively affect families, which implies the need for adequate resources, time availability, and support so that these professionals can effectively perform their educational role [20, 22]. Furthermore, access to educational technologies for care continuity, such as booklets, videos, apps, and telehealth, can be useful to provide additional support and skills to family members in home care [24, 25]. Preparing for the transition from hospital to home involves education and training for parents/caregivers and organizing a clinical follow-up plan. Despite the challenges involved in planning for these transitions, achieving a reduced hospital stay and providing the child with a quality life at home surrounded by the support of their family are essential goals and should be prioritized [26].

Family members of children undergoing cancer treatment who participated in this study emphasized the importance of having their individual educational and care needs met during the transition process. These needs are reflected in educational demands on how to care for the child at home after diagnosis, especially care for the long-term catheter, feeding at home, as well as guidance on the disease and daily life after the start of treatment. Thus, adaptation of the guidelines shared by nurses must be in accordance with the social, educational, cultural, and economic reality of the family members [21]. This implies a sensitive, horizontal approach centered on dialog, recognizing the autonomy and uniqueness of each party involved [25].

The hospital-to-home transition is a delicate phase marked by the need for continuous use of long-term catheters, as they are essential devices for administering chemotherapy, blood components, and antimicrobials [27, 28]. This situational transition requires facing challenges related to catheter care at home, as its presence and the side effects of chemotherapy can temporarily limit the child's social activities and lifestyle, such as riding a bike, running, jumping, sunbathing, and swimming [28–31]. The diagnosis of childhood cancer causes significant changes in daily life, requiring adaptation strategies to deal with this new reality.

In addition to restrictions on play, children's diets also undergo changes, as reported in this study. These changes are due to neutropenia from chemotherapy characterized by decreased immunity, which can lead to risks of infections and worsening of diseases [32, 33]. Therefore, it is essential to carefully wash fruits and vegetables immediately before consumption, opt for baked or cooked foods, and avoid eating raw foods to ensure food safety [34].

Foods of unknown origin and handling should also be avoided, preferring those with individual packaging for single consumption, and dairy products should only be used if they are pasteurized. Water for consumption should be of mineral origin and bottled; otherwise, it should be boiled. These precautions aim to ensure a diet free of microorganisms, contributing to prevent infections [33, 35]. This transitional process is marked by changes in eating habits and routines, and requires awareness and engagement of the entire family in facing a critical event that, in this case, is related to childhood cancer as a determinant of restrictions, such as food, social activities, and lifestyles [12].

Two family members participating in this study reported poor acceptance of the new eating plan required due to cancer and chemotherapy, which increases the risk of malnutrition. Malnutrition is a substantial concern among children diagnosed with cancer, with rates estimated at around 75%. This condition emphasizes the need for adequate nutritional support during treatment to improve therapeutic outcomes [35]. Treatment-associated eating disorders, such as food refusal and decreased intake, are also common and require special attention from health professionals [36].

Although many of the stressors associated with cancer diagnosis and treatment are unchangeable, family rules and routines are modifiable behaviors capable of strengthening family skills to cope with and adapt to the new reality [37]. It is understood that such conditions require family members to not only acquire knowledge but also modify behaviors and redefine their identities. This phenomenon corroborates a situational transition, in which the nurse performs interventions that help family members to go through this process and develop healthy response patterns [12, 38].

Finally, it is observed that the care of children undergoing treatment for hematologic cancer requires a holistic approach beyond their clinical needs, which includes the family context, the emotional dynamics involved, and the need to systematize actions aimed at improving the care process. Such improvement is achieved through adopting practices based on responsibility, sensitivity, creativity, ethics, empathy, active, and open listening skills, in addition to the ability to express feelings without judgment or censorship. It is therefore essential to adapt care individually to the needs of children and their families who experience a cancer diagnosis during the hospital–home transition.

### 4.1. Study Limitations

The study was limited to a single public pediatric hospital in the city of Rio de Janeiro (Brazil), which may not reflect the reality of other institutions or regions, limiting generalization of the results. Although participant selection was careful, it excluded family members of children in palliative care, which may have left out important perspectives. Therefore, the conclusions of the study may not be applicable in different or broader contexts.

### 4.2. Implications for Clinical Practice

The findings of this study highlight the need for specific educational programs for family members, but also for health professionals themselves, especially nurses, in order to provide effective care and reduce the risk of complications and hospital readmissions. The study also suggests integrating psychological and social support services for family members in order to better cope with the stress and anxiety associated with treatment and the transition home. Such implications may contribute to care continuity, improving quality of life and clinical outcomes.

## 5. Conclusion and Recommendations

It is concluded that the educational and care demands of family members of children undergoing hematological oncology treatment during the hospital–home transition include the need for detailed and written guidance on various aspects of home care, such as medication administration, catheter care, hygiene procedures, and adequate nutrition during treatment, as well as the need for support to deal with constant concerns, questions and uncertainty about the disease, symptom management, and adverse reactions to treatment.

Nurses must adopt careful and detailed planning to meet the educational and care demands of family members of children with hematologic cancer during the hospital–home transition. This planning must be based on practices that promote continuous dialog, sharing clear guidelines, including with the support of effective educational technologies. Such strategies are essential to help families adapt to the individual and specific needs of their children, ensuring a safer and smoother transition to the home environment.

The use of educational measures and technologies (printed materials, videos, mobile applications, podcasts, websites, and other learning platforms) is a strategic point in training these families in the sense that they can develop skills on how to provide safe and effective care at home, such as medication administration techniques, care for catheters and other clinical devices, monitoring vital signs, identifying signs and symptoms of complications, strategies for managing stress and anxiety, among other aspects. This interaction between family members and nurses based on collaboration also enables identifying, clarifying, and recognizing the meanings, behaviors, and effects resulting from the process experienced.

Nurses' activities in health education are therefore an essential tool for creating spaces for new knowledge construction, strengthening interpersonal relationships, and effective dialog. In this sense, it is strategic for this professional to be attentive to the educational demands of families, recognizing their difficulties and promoting a collaborative and welcoming learning environment, thereby enabling a healthy hospital–home transition.

## Data Availability

Data will be made available by the authors upon request.
